# The effects of amoxicillin treatment of newborn piglets on the prevalence of hernias and abscesses, growth and ampicillin resistance of intestinal coliform bacteria in weaned pigs

**DOI:** 10.1371/journal.pone.0172150

**Published:** 2017-02-15

**Authors:** Jinhyeon Yun, Satu Olkkola, Marja-Liisa Hänninen, Claudio Oliviero, Mari Heinonen

**Affiliations:** 1 Research Centre for Animal Welfare, Department of Production Animal Medicine, Faculty of Veterinary Medicine, University of Helsinki, Helsinki, Finland; 2 Antibiotics Section, Food and Feed Microbiology Research unit, Research and Laboratory Department, Finnish Food Safety Authority Evira, Mustialankatu 3, Helsinki, Finland; 3 Department of Food Hygiene and Environmental Health, Faculty of Veterinary Medicine, University of Helsinki, Helsinki, Finland; 4 Department of Production Animal Medicine, Faculty of Veterinary Medicine, University of Helsinki, Paroninkuja 20, Saarentaus, Finland; University of Connecticut, UNITED STATES

## Abstract

This study investigated the effects of a single amoxicillin treatment of newborn piglets on the prevalence of hernias and abscesses until the age of nine weeks. We also studied whether the treatment was associated with growth and mortality, the need for treatment of other diseases, the proportions of ampicillin resistant coliforms and antimicrobial resistance patterns of intestinal *Escherichia coli* (*E*. *coli*). A total of 7156 piglets, from approximately 480 litters, were divided into two treatment groups: ANT (N = 3661) and CON (N = 3495), where piglets were treated with or without a single intramuscular injection of 75 mg amoxicillin one day after birth, respectively. The umbilical and inguinal areas of weaned pigs were palpated at four and nine weeks of age. At the same time, altogether 124 pigs with hernias or abscesses and 820 non-defective pigs from three pens per batch were weighed individually. Mortality and the need to treat piglets for other diseases were recorded. Piglet faecal samples were collected from three areas of the floors of each pen at four weeks of age. The prevalence of umbilical hernias or abscesses did not differ between the groups at four weeks of age, but it was higher in the CON group than in the ANT group at nine weeks of age (2.3% vs. 0.7%, *P* < 0.05). Numbers of inguinal hernias and abscesses did not differ between the groups at four or nine weeks of age. The ANT group, when it compared with the CON group, increased the weight gain between four and nine weeks of age (LS means ± SE; 497.5 g/d ± 5.0 vs. 475.3 g/d ± 4.9, *P* < 0.01), and decreased piglet mortality (19.5% ± 1.0 vs. 6.9% ± 1.0, *P* < 0.05) and the need to treat the piglets for leg problems (3.4% ± 0.3 vs. 1.9% ± 0.3%, *P* < 0.01) but not for other diseases by the age of four weeks. The proportion of ampicillin resistant intestinal coliform bacteria and the resistance patterns of the *E*. *coli* isolates were not different between the ANT and CON groups. In conclusion, our results showed that the amoxicillin treatment of new-born piglets produced statistically significant effect in some of the parameters studied. However, as these effects were only minor, we did not find grounds to recommend preventive antibiotic treatment. Further, continuous antimicrobial treatment of newborn piglets could negatively influence the development of the normal microbiota of the piglet and promote selection of antimicrobial resistance genes in herds. Therefore we suggest rejection of the use of routine administration of antimicrobial agents at birth.

## Introduction

Pigs with hernias in commercial herds are usually culled or sold for a low price [1], and associated with lower growth rates and higher mortality rate than unaffected group mates [2]. Thus, hernias apparently result in economic losses and welfare concerns in modern swine farming.

The prevalence of hernias in piglets varies according to gender, breed and environment. Porcine umbilical hernias are mainly due to incomplete closure of the umbilical cord after birth, which is caused by genetic variability in musculature of the navel cord or infection of the umbilical stump or both [1, 3]. Inguinal and scrotal hernias are defined by the protrusion of the hernia contents into the inguinal canal or scrotum. It is known that most inguinal hernias are influenced by hereditary factors [4, 5, 6, 7].

Despite preventive antimicrobial treatment not being recommended in Finland, some farmers administer antimicrobials to newborn piglets in order to reduce the prevalence of umbilical infections and hernias. However, previous studies showed that administration of oxytetracycline to newborn piglets failed to reduce mortality and several diseases, except foot abscesses, until death [8], or had no effect on development of umbilical hernias [1]. In addition, Rutten-Ramos and Deen [5] reported that administration of long-acting ceftiofur to piglets at birth did not affect hernia prevalence. Only anecdotal evidence is available regarding the effectiveness of using antimicrobials to prevent umbilical hernias, and the reports do not appear in peer-reviewed journals [9, 10]. However, studies on humans [11, 12] and pigs [13] showed that the use of antibiotics for newborns disturbs normal development of microbiota in the gastrointestinal tract for a considerable time and the use of antimicrobials lead to an increase in drug resistance of the normal microbiota [14, 15].

Finnish national monitoring data for indicator *Escherichia coli (E*. *coli)* from faeces of domestic pigs collected at abattoirs indicate that the most common resistances are against tetracycline (TET), streptomycin (STR), trimethoprim (TM), sulfamethoxazole (SU) and ampicillin (AMP) (www.zoonoosikeskus.fi). These resistance traits have also been common in *E*. *coli* from pigs in other countries [16]. Moreover, in a previous study, ampicillin resistant *E*. *coli* were found to be mainly multi-resistant, being commonly resistant also to chloramphenicol (CM), TET, STR, SU and TM [17].

The use of antimicrobials is required in pig production for treating diseased animals. However, prophylactic administration of antimicrobials directly after birth should be considered carefully. Consequently we aimed to investigate the effects of a single amoxicillin treatment of newborn piglets on the prevalence of hernias and abscesses in the umbilical and inguinal areas until the age of nine weeks. We also aimed to study whether the treatment is associated with growth, mortality, or the need to treat piglets for other diseases. We hypothesised that a single preventive dose of amoxicillin to newborn piglets does not influence the prevalence of hernias, but it may have an effect on the need to treat piglets for other diseases, especially intestinal disorders. Finally, we aimed to determine whether amoxicillin treatment of newborn piglets increases the prevalence of aminopenicillin resistant coliforms and characterize the resistance patterns of faecal *E*. *coli* isolates.

## Materials and methods

The study procedure was reviewed and approved by the Ethical Committee for Institutional Animal Use and Care of the University of Helsinki. The experiment was conducted in a commercial pig farm in southern Finland from August 2015 to January 2016.

### Animals and management of the herd

The study herd was of approximately 850 sows (Landrace × Yorkshire). Sows and gilts (hereafter sows) were moved to a farrowing pen between three and eight days before parturition, and kept there until weaning. The pen consisted of a conventional steel farrowing crate on a half-slatted concrete floor with the piglet shelter situated in one corner with a heat lamp on a solid concrete floor. A bucketful of sawdust was provided daily on the solid floor. Sows were allowed *ad libitum* access to water from a nipple drinker and were fed a standard lactation diet three times a day via an automatic dry feeding system. A creep feeding diet (NE 11.0 Mj/kg; CP 19.0%) for the nursery piglets was provided after 14 days of birth until weaning.

The umbilical area of newborn piglets was not disinfected after birth. Umbilical cords longer than 12 cm were cut at that length after drying-up during the first day of life. The piglets were cross-fostered to even up the litters at two days after birth at the latest. At the age of two to five days, the farm personnel routinely picked up each piglet individually for an intramuscular injection of iron, 200 mg (Ursoferran^®^, 200 mg/ml) and per oral dosing of coccidiostat toltratsuril, 20 mg/kg (Toltarox^®^, 50 mg/ml). During the same handling, male piglets were given an intramuscular injection of meloxicam, 0.4 mg/kg (Metacam^®^, 5 mg/ml) and castrated. The teeth of the piglets were not routinely polished, except for those biting the udders of the sow noticeably. Tails of the piglets were not docked.

Piglets were weaned at approximately 28 ± 3 days and vaccinated against circovirus about one week after weaning with 1 ml of intramuscular Ingelvac Circoflex^®^. Pigs were transported to a temperature-controlled room at weaning. Each room had 20 pens with approximately 20 to 25 pigs per pen (2.5 × 5.0 m). The pens were thoroughly cleaned, disinfected and dried before piglets were transported. Each pen had a solid concrete floor with 25% of slatted dung area and was provided with three bucketfuls of sawdust. After pigs moved in, a supplementary shovelful of sawdust per day was added on the ground if it was soiled or dissipated. Pigs in each pen were allowed ad libitum access to water from three nipple drinkers and fed a standard weaning diet (NE 10.5 Mj/kg; CP 17.5%) for 10 days, and a growing diet (NE 9.9 Mj/kg; CP 16.5%) for 28 days subsequently.

### Experimental design and data collection

A total of 480 litters (12 batches, approximately 40 litters in one batch where sows were giving birth within one week), producing 7156 piglets, were assigned to two groups during the first day after birth. A coin was flipped for the first batch to allocate the treatment, ANT (amoxicillin treatment) or CON (non-treatment). Subsequently every other batch was allocated to ANT (6 batches) or CON (6 batches). The farm personnel lifted each piglet on to a trolley for medication and ear-tagging: 1) ANT—A total of 3661 pigs from 240 litters in six batches were given an individually numbered green ear tag on their left ear and injected with 75 mg of amoxicillin (0.5 ml of Vetrimoxin®, vet 150 mg/ml, Ceva Sante Animale) in their neck muscle, 2) CON—A total of 3495 pigs from the other 240 litters in six batches were given an individually numbered orange ear tag on their right ear and not treated with any routine antibiotics at birth. Cross-fostering was allowed only within the same treatment group. At weaning, the piglets in the same treatment groups were housed in the same pens. The researchers involved in evaluations and statistical analyses were unaware of the allocation of the piglets to the different groups during the entire sampling periods and statistical analyses.

The farm personnel treated piglet diseases according to the advice of the health care veterinarian of the herd. They recorded the ear-number of the treated piglets as well as the dates and reasons for treatments on a sow card hanging above the farrowing pen. In general, amoxicillin was used to treat leg problems and skin infections of suckling piglets, and dihydrostreptomycin was used for the treatment of diarrhoea. The farm personnel stored the ear-tags of all euthanized or dead piglets daily in a plastic bag marked with the date. The researchers collected the bags for recording mortality until weaning.

The first author palpated the umbilical and inguinal areas of the pigs twice while the animals were standing to diagnose the presence of hernias and abscesses, approximately three (Total N = 6523) and 38 days (Total N = 6451) after weaning, i.e. at about four and nine weeks of age (hereafter four and nine weeks, respectively). The only reason for the drop in the numbers of animals included in the present study was that the animals died between birth and nine weeks. An umbilical hernia was determined as a discontinuity of the abdominal wall at the umbilicus with protrusion of abdominal content into a hernia sac formed by the skin and surrounding connective tissue [18]. An inguinal hernia was defined as a condition where intestines or other abdominal organs pass into the inguinal canal through an abnormally large and patent vaginal orifice through which the vaginal process and peritoneal cavity communicate [18]. An abscess was recorded when an enclosed collection of pus with a firm formation was palpated. The length of hernia ring as well as the diameter of the hernia and the abscess was estimated in centimetres. When both hernia and abscess were present, their joint diameter was measured.

At the same time points, i.e. four and nine weeks, three pens per batch (altogether 36 pens with 408 ANT and 415 CON pigs) were consistently selected from the middle of the room and each individual pig with no defects in umbilical and inguinal areas (hereafter non-defective pigs) from the pens was weighed. Also, the body weights of pigs diagnosed with a hernia, abscess or both were recorded individually on the same days.

Pen hygiene was evaluated by a visual inspection. The levels were scored as 0: no visual contamination was observed, 1: less than 25% of the pen was contaminated with manure, 2: more than 25% and less than 50% of the pen was contaminated with manure, 3: more than 50% and less than 75% of the pen was contaminated with manure, and 4: more than 75% of the pen was contaminated with the pen.

### Faecal sampling and antimicrobial susceptibility testing

#### Sample collection and cultivation

Approximately three days after weaning, in a total of ten batches, approximately 5 g of fresh faeces were collected from three to four areas of the floors of each pen. All faecal samples from the same pen were pooled into a single transport cup yielding altogether 102 pooled samples (48 from the ANT and 54 from the CON group). The samples were placed in ice-chilled boxes and transported to the laboratory on the same day. Samples were kept overnight at +4°C and cultivated on the following day.

From each sample, 1 g of faecal material was weighed and suspended in 10 ml of peptone water in a stomacher filter bag. The samples were then homogenized and filtered for 60 s using a Stomacher paddle blender 400 (www.seward.co.uk). In order to determine the colony forming units (CFUs) for total coliforms and the percentage of AMP-resistant coliform population in each sample, tenfold serial dilutions (dilution ratios 1:10–1:100 000) were prepared in peptone water and 100 μl of the appropriate dilutions were spread on MacConkey agar plates (Merck, Darmstadt, Germany) with and without 10 mg/l ampicillin, respectively. The plates were incubated overnight at 37°C and the numbers of lactose-fermenting bacteria (red colonies, referred to as coliforms according to the definition of coliform bacteria) were counted from plates containing between 10 and 200 colonies.

For each sample, two typical distinct *E*. *coli* colonies (indicated by red colour and bile salt precipitation halo) were picked and subcultivated on Luria Bertani (LB) agar plates (Difco, Sparks, MD) and incubated overnight. These colonies were confirmed to be *E*. *coli* by their ability to convert tryptophan to indole and by the ability to produce ß-galactosidase (yellow colour) and ß-glucuronidase (fluorescent under UV-light) (Colilert-18, IDEXX Laboratories, Maine, USA). Two confirmed *E*. *coli* colonies from each sample, one originating from non-selective MacConkey and the other from MacConkey with 10 mg/l AMP, were stored for further testing at -70°C in nutrient broth with 15% glycerol.

#### Susceptibility testing

The susceptibility (minimum inhibitory concentration, MIC) of the confirmed *E*. *coli* isolates was tested against AMP, ciprofloxacin (CIP), nalidixic acid (NAL), gentamicin (GEN), STR, TET, florfenicol (FF), colistin (CS), SU, TM, CM, kanamycin (KAN), cefotaxime (CTX) and ceftazidime (CAZ) using the broth microdilution method (VetMIC GN-mo, ver. 4, National Veterinary Institute, Uppsala, Sweden) according to manufacturer’s instructions. *E*. *coli* type strain ATCC 29522 was used as a control. The *E*. *coli* epidemiological cut-off values (ECOFFs), determined by the European Committee on Antimicrobial Susceptibility Testing (EUCAST, www.eucast.org), were applied to differentiate between wild type (also denoted as susceptible) and non-wild type (also denoted as resistant) populations.

#### Screening for the *mcr-1* gene

All *E*. *coli* isolates with CS MIC ≥ 2 mg/l were screened for the presence of the *mcr-1* gene, a plasmid-mediated colistin resistance conferring gene [19], at the Finnish Food Safety Authority Evira using the DTU PCR protocol [20] with some modifications as follows. PCR was performed using DreamTaq DNA-polymerase and 10 x DreamTaq buffer (Thermo Fischer Scientific, Waltham, MA, USA) and amplification of the 16S rRNA gene fragment was used as an internal control with previously described primers [21]. The cycling conditions were 94°C for 15 s, 58°C for 1 min and 72°C for 1 min, repeated 25 times. As a positive control, *E*. *coli* strain EURL 0412016126 harbouring *mcr-1* was used in the assay.

### Statistical analysis

SAS v.9.4 (SAS Institute Inc., NC, USA, 2012) was used for statistical processing of the data for hernia occurrence, mortality and body weight. Significant differences between antimicrobial and control treatments were tested using the Tukey-Kramer procedure. A mixed model was fitted to the data for analysis of the occurrence ratio and the size of hernias using batch as a random effect. The pen was the experimental unit and the individual animal was the sample unit for analysis of the occurrence ratio of hernias. The individual animal was the experimental unit for analysis of hernia size. Piglet mortality ratio and the ratio of piglets needing treatment for other diseases were analysed with a fixed effect one-way ANOVA model using PROC GLM. The mixed procedure was fitted to the body weight data for non-defective pigs. The experimental unit was the pen for analysis of body weights of pigs selected in three pens per batch, or the individual animal for analysis of body weights of pigs diagnosed with defects. The level of pen hygiene was analysed with a mixed model and tested to determine interactions with incidence of hernias and abscesses by Spearman rank correlation coefficients.

SPSS for Windows ver. 22 (IBM Corp., Armonk, NY, USA) was used for data processing and statistical analyses of the coliform and *E*. *coli* prevalence and resistance data. The Saphiro-Wilk test was used to test for normality of coliform prevalence and proportions of AMP-resistant coliforms. The Mann-Whitney U test was used to assess the difference between treatments in the prevalence of total coliforms and their fraction of AMP resistance. A Pearson Chi-square test was used to test the significance of the associations between different resistance traits. Logistic regression using binary logistics was used to assess the probability of an *E*. *coli* being multidrug resistant (MDR; i.e., simultaneous resistance to ≥ 3 antimicrobial classes) using AMP susceptibility and treatment group membership as predictors. Goodness of fit was tested using the Hosmer and Lemeshow test, and the reference categories for each predictor were chosen to ease the interpretation of the odds ratios. Only the results from regression analysis, in which the test of the full model versus the model with an intercept was statistically significant, were reported. Significant difference was set at *P* < 0.05.

## Results

### Occurrence and size of hernias, abscesses or both

Out of a total of 6523 pigs palpated at four weeks, umbilical hernias or abscesses were found from three [0.05%, (2 barrow and 1 female)], or eight [0.1%, (2 barrow and 6 female)] pigs, respectively. At the same time point, inguinal hernias, abscesses or both were found from six [0.1%, (5 barrow and 1 female)], 20 (0.3%, all barrow), or two (0.03%, all barrow) pigs, respectively. The occurrence ratio of umbilical or inguinal hernias, abscesses or both was not significantly different between the ANT and CON groups palpated at four weeks.

Out of a total of 6451 pigs palpated at nine weeks, umbilical hernias, abscesses or both were found from 30 [0.5%, (17 barrow and 13 female)], 43 [0.7%, (20 barrow and 23 female)], or 22 [0.3%, (13 barrow and 9 female)] pigs, respectively. At the same time point, inguinal hernias or abscesses were found from seven (0.1%, all barrow), or 17 [0.3%, (16 barrow and 1 female)] pigs, respectively. The occurrence ratio of umbilical hernias, abscesses or both in the ANT groups was significantly lower than in CON groups (*P* < 0.05 for all, Table 1). However, the ratio of inguinal hernias, abscesses or both was not significantly different between the ANT and CON groups at nine weeks of age (Table 1).

**Table 1:** Effects of amoxicillin (ANT) or no (CON) treatment for newborn piglets on occurrence ratio and the size of hernias, abscesses or both in umbilical and inguinal areas at nine weeks of age ^1^.

		N		Occurrence ratio, % ^2^		Size, *cm* ^3^		P value	
		ANT	CON	ANT	CON	ANT	CON	ratio	Size
Umbilical	Hernia	8	22	0.2 ± 0.1	0.7 ± 0.1	9.5 ± 5.7	8.5 ± 7.1	0.02	0.74
	Abscess	16	27	0.4 ± 0.2	1.0 ± 0.2	2.8 ± 2.4	2.0 ± 1.4	0.02	0.16
	Hernia + Abscess,	5	17	0.1 ± 0.2	0.6 ± 0.2	7.4 ± 7.7	6.8 ± 3.3	0.02	0.82
	Total	29	66	0.7 ± 0.2	2.3 ± 0.2	**-**	**-**	< 0.0001	**-**
Inguinal	Hernia	4	3	0.1 ± 0.1	0.1 ± 0.1	9.5 ± 1.9	16.0 ± 5.3	0.94	0.07
	Abscess	10	7	0.4 ± 0.1	0.2 ± 0.1	2.6 ± 1.8	3.1 ± 2.2	0.19	0.63
	Hernia + Abscess,	0	0	-	-	-	-	-	-
	Total	14	10	0.5 ± 0.1	0.3 ± 0.1	-	-	0.23	-

^1^ Weaned pigs (Total N: ANT = 3434, CON = 3017) were palpated at nine weeks after birth (i.e. approximately three and 38 days after the weaning, respectively).

^2^ Values represent least squares (LS) means ± SE of the occurrence ratio of hernias, abscess or both.

^3^Values represent means ± SD of the size of hernias, abscess or both.

The size of umbilical and inguinal hernias, abscesses or both was not significantly different between the ANT and CON groups or between barrow and female pigs at both four and nine weeks of age. Of the 39 pigs that were diagnosed with umbilical or inguinal hernias, abscesses or both at four weeks of age, 19 (49%) pigs still showed the same defects while these defects were not detected from the remaining animals at the age of nine weeks. The means of changes ± SD in size of the defects between four and nine weeks of age from the 19 pigs were as follows: umbilical hernias (N = 6), 9.3 cm ± 8.8, umbilical abscesses (N = 3), 4.5 cm ± 4.3, inguinal hernias (N = 6), 6.0 cm ± 3.6, and inguinal abscesses (N = 4), - 1.1 cm ± 6.0.

### Body weight and mortality

**Fig 1:**
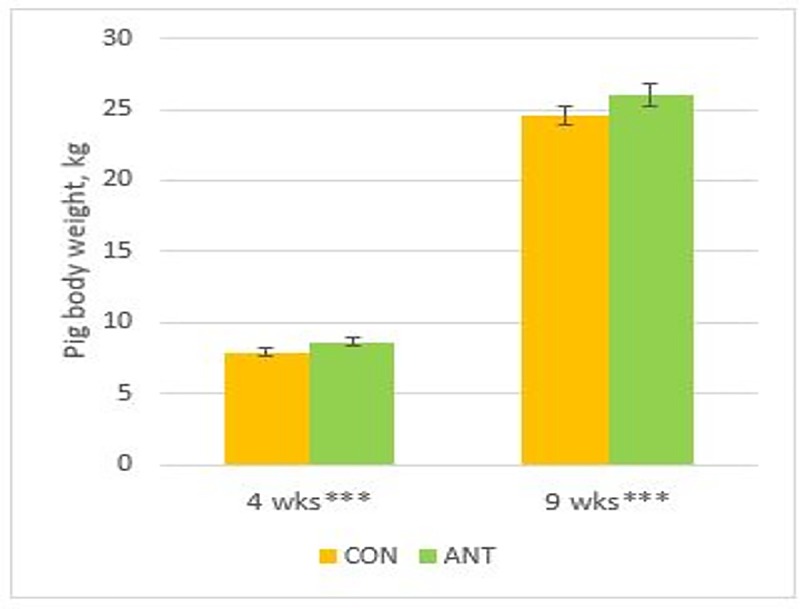
Body weight of 820 pigs with no palpable defects in umbilical or inguinal area at four and nine weeks of age. The pigs were treated with a single amoxicillin injection one day after birth (ANT, N = 405), or not treated (CON, N = 415). Asterisks (***) indicate that variables were significantly different (*P* < 0.0001).

Average body weight measured from a total of 820 non-defective pigs was 8.2 kg ± 1.8 and 25.3 kg ± 4.6 at the age of four and nine weeks, respectively. The average body weight of 36 pigs diagnosed with hernias, abscesses or both at four weeks of age was 7.9 kg ± 2.1, and that of 88 pigs at nine weeks of age was 25.1 kg ± 5.2. The treatment did not have a significant effect on body weights of pigs with hernias, abscesses or both at the ages of four or nine weeks. However, the average body weights of non-defective pigs in the ANT groups were significantly higher than those in the CON groups at four and nine weeks of age (*P* < 0.0001, for both, Fig 1).

**Fig 2:**
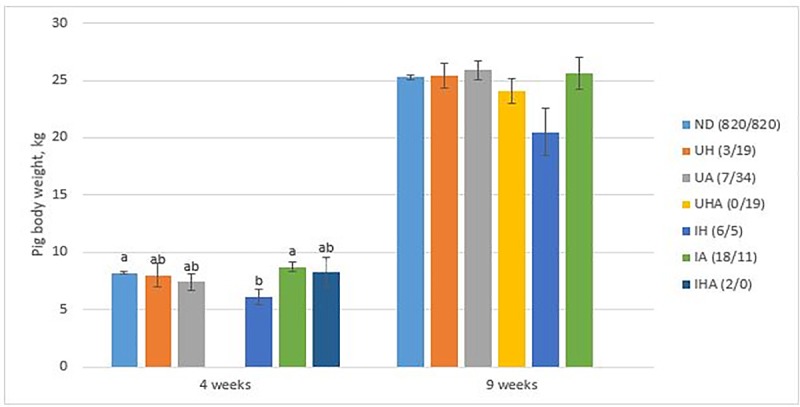
Body weight of pigs with umbilical hernia (UH), umbilical abscess (UA), umbilical hernia + abscess (UHA), inguinal hernia (IH), inguinal abscess (IA), inguinal hernia + abscess (IHA), or with no defects (ND). The numbers in the figure represent the number of affected pigs (four weeks/nine weeks). Different letters (a, b) indicate that there were significant differences between variables (*P* < 0.05).

The average body weight of pigs that had an inguinal hernia (N = 6) was lower than that of pigs with an inguinal abscess (N = 18) or non-defective pigs (N = 820) at four weeks of age (*P* < 0.05, Fig 2). However, body weights of pigs with different defects were not significantly different regardless of the types of hernia or abscess at nine weeks (Fig 2).

The average daily weight gain of the non-defective pigs between weeks four and nine was greater in the ANT groups (N = 405) compared with the CON groups (N = 415) (LS means ± SE; 497.5 g/d ± 5.0 vs. 475.3 g/d ± 4.9, *P* < 0.01). Mean ± SD of the average daily gain between weeks four and nine in pigs was 432.1 g/d ± 91.1 for an umbilical hernia (N = 6), 385.7 g/d ± 210.4 for an umbilical abscess (N = 3), 361.9 g/d ± 90.2 for an inguinal hernia (N = 6), and 460.7 g/d ± 105.9 for an inguinal abscess (N = 4).

**Fig 3:**
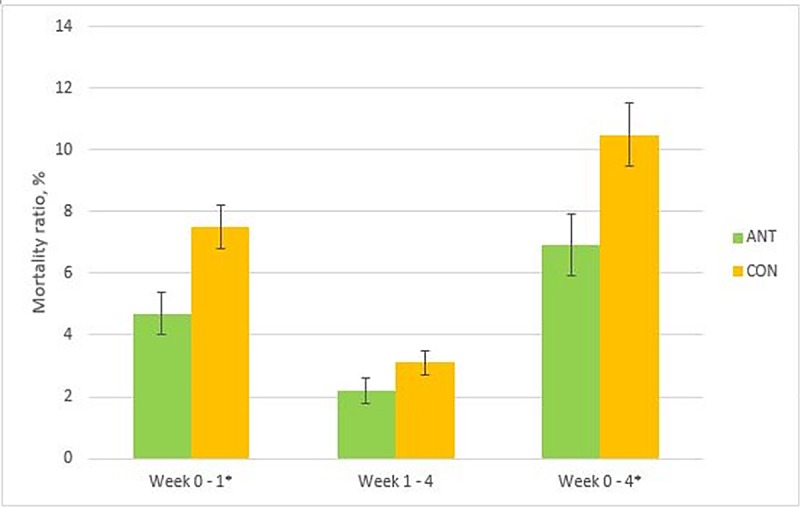
Mortality of pigs (total N = 7156) from birth to the first week (Week 0–1), from the first to the fourth week (Week 1–4), and from birth to the fourth week (Week 0–4). The pigs were treated with a single amoxicillin injection on the first day after birth (ANT, N = 3661), or not treated (CON, N = 3495). An asterisk (*) indicates that variables were significantly different (*P* < 0.05).

The piglet mortality ratios in the CON group were higher than those in the ANT group during the first week of life (LS means ± SE; 7.5% ± 0.7 vs. 4.7% ± 0.7) and between birth and fourth week (10.5% ± 1.0 vs. 6.9% ± 1.0) (*P* < 0.05 for both, Fig 3). Two deaths out of 39 pigs with hernias, abscesses, or both at weaning occurred in the CON group during the entire experimental period.

### Treatments for other diseases

The piglets in the CON group had a higher percentage of leg problems than the piglets in the ANT group (*P* < 0.01, Table 2). The occurrence ratio of pigs needing treatment for other diseases was not significantly different between CON and ANT groups during the nursing period (Table 2). The most common medicine used for treating sick piglets during the nursing period in this study herd was amoxicillin.

**Table 2:** The occurrence ratio of piglets treated for different diseases during the suckling period after routine amoxicillin injection during the first day of life (ANT) or no treatment (CON).^1^

	N		Occurrence ratio, %		
	ANT	CON	ANT	CON	*P* value
Indications for the treatment, %					
Leg problems	70	118	1.9 ± 0.3	3.4 ± 0.3	< 0.01
Diarrhoea	60	58	2.0 ± 1.1	1.5 ± 1.1	0.77
Skin problems	8	20	0.2 ± 0.2	0.6 ± 0.2	0.15
Any diseases ^2^	159	250	4.6 ± 1.2	7.0 ± 1.2	0.18
Medicines used for the treatments, %					
Amoxicillin	140	241	3.7 ± 1.2	6.9 ± 1.2	0.08
Dihydrostreptomycin	48	19	1.7 ± 0.9	0.5 ± 0.9	0.40
Any medications ^3^	197	274	5.6 ± 1.3	7.7 ± 1.3	0.26

^1^ Values represent LS means ± SE of the occurrence ratio of the piglets (Total N: ANT = 3661, CON = 3495) treated with additional antibiotics.

^2^ Any diseases include leg, skin and eye problems, diarrhoea, weak piglets, and undefined diseases.

^3^ Any medications include amoxicillin, dihydrostreptomycin, cortisone, meloxicam, procainpenicillin and other antimicrobials (not specified).

### The association with the pen hygiene levels

The levels of pen hygiene were not different between the ANT (1.0 ± SE 0.1) and CON (1.0 ± SE 0.1) groups, and had no effect on occurrence of hernias or abscesses, body weight, and mortality of pigs during the experimental period.

### Antimicrobial susceptibility of intestinal coliforms and *E*. *coli*

#### Prevalence of coliform bacteria

We were able to determine the total coliform counts from 52 pooled fecal samples from the ANT group and 45 fecal samples from the CON group. The coliform prevalence data in both groups were non-normally distributed. The median of the CFUs were 6.0 x 10^6^ (range 1.2 × 105–1.5 × 10^8^)/g faeces and 5.9 x 10^6^ (range 2.1 × 10^5^–6.0 × 10^7^)/g feces in the samples originating from the CON and ANT groups respectively. No statistically significant difference in the prevalence of coliforms was found between the groups.

#### Proportions of AMP resistant coliforms

The proportions of AMP resistant colonies (MIC > 8 mg/l) were calculated from 48 and 39 samples in the ANT and CON groups, respectively. A non-normal distribution was evident also for these data in both groups. The median percentages of AMP-resistant coliforms were 31.0% and 26.6% and the prevalence ranged between 1–100% and 2–74% in the CON and ANT groups, respectively. The values between the groups were not significantly different.

#### MIC determination and associations between resistance traits

Altogether, 111 confirmed *E*. *coli* isolates (52 isolates from AMP-selective plates and 59 from plates without AMP and 50 and 61 isolates originating from ANT and CON groups, respectively) from a total of 60 faecal samples were tested. All isolates from selective MacConkey plates containing 10 mg/l AMP and 20/59 (33.9%) of *E*. *coli* isolates from MacConkey plates without AMP were AMP-resistant to AMP MICs ≥ 64 mg/l. The proportions of resistant isolates against each of the studied antimicrobials in both treatment groups originating from AMP-selective and non-selective MacConkey agar plates are shown in Table 3. In both groups, resistances against AMP, STR, TET, SU and TM were most commonly observed pattern.

**Table 3:** Percentages of resistant *E*. *coli* isolates (N = 111) from faeces of weaned pigs isolated on MacConkey plates with and without 10 mg/l ampicillin (AMP).

Antimicrobials^2^	No AMP in plate		AMP in plate		FinresVet 2013^1^, % (N = 315)
	ANT, % (N = 26)	CON, % (N = 33)	ANT, % (N = 24)	CON, % (N = 28)	
AMP	34.6	33.3	100	100	9.5
CIP	3.8	6.1	29.2	28.6	1.9
NAL	-	-	4.2	-	1.3
GEN	-	3	-	-	1
STR	46.2	51.5	83.3	89.3	18.4
FF	-	-	-	-	1
TET	53.8	51.5	70.8	71.4	23.5
CS	-	-	-	3.6	-
SU	46.2	45.5	66.7	67.9	13.7
TM	46.2	45.5	58.3	67.9	12.4
CM	3.8	-	-	3.6	1
KAN	7.7	6.1	8.3	-	2.9
CTX	-	-	-	-	0.6
CAZ	-	-	-	-	-

Pigs in ANT group were treated with amoxicillin during the first day of life while CON pigs were not.

^1^ Data originating from the national monitoring program of the Finnish Food Safety Authority for resistance in indicator *E*. *coli* from swine faeces collected at abattoirs in 2013 (www.zoonoosikeskus.fi).

^2^ AMP = ampicillin, CIP = ciprofloxacin, NAL = nalidixic acid, GEN = gentamicin, STR = streptomycin, FF = florfenicol, TET = tetracycline, CS = colistin, SU = sulfamethoxazole, TM = trimethoprim, CM = chloramphenicol, KAN = kanamycin, CTX = cefotaxime, CAZ = ceftazidime.

Altogether 23 resistance profiles were found among the tested isolates (Table 4). All AMP-resistant *E*. *coli* colonies were resistant to at least one other antimicrobial with the most common resistance pattern being AMP-STR-TET-SU-TM (N = 38), which was the most common among the isolates from AMP-selective plates in both groups (41.7% in the ANT and 53.6% in the CON group). Susceptibility to all the studied antimicrobials (42.3% and 36.4% in the ANT and CON groups, respectively) was the most common profile in both groups among isolates obtained through non-selective isolation, followed by AMP-STR-TET-SU-TM (Table 4).

**Table 4:** Resistance profiles of *E*. *coli* isolated on MacConkey with and without 10 mg/l ampicillin (AMP) and their counts.

	No AMP in plate		AMP in plate	
Resistance profile^1^	ANT, n (N = 26)	CON, n (N = 33)	ANT, n (N = 26)	CON, n (N = 33)
Susceptible to all antimicrobials tested	11	12	-	-
AMP-CIP	-	1	2	1
AMP-CIP-CS	-	-	-	1
AMP-CIP-NAL-STR-SU-TM-KAN	-	-	1	-
AMP-CIP-STR	-	1	3	4
AMP-CIP-STR-SU-TM	1	-	-	1
AMP-CIP-STR-TET-SU-TM	-	-	1	1
AMP-STR	1	-	-	-
AMP-STR-SU-TM	-	-	1	1
AMP-STR-TET	-	-	2	2
AMP-STR-TET-SU	-	1	1	-
AMP-STR-TET-SU-TM	5	8	10	15
AMP-STR-TET-SU-TM-CM	1	-	-	1
AMP-STR-TET-SU-TM-KAN	1	-	1	-
AMP-TET	-	-	1	-
AMP-TET-SU	-	-	1	-
AMP-TET-SU-TM	-	-	-	1
GEN-STR	-	1	-	-
STR-KAN	1	-	-	-
STR-TET-SU-TM	4	6	-	-
TET	1	-	-	-
TET-KAN	-	2	-	-
TM	-	1	-	-

Pigs in ANT group were treated with amoxicillin during the first day of life while CON pigs were not.

^1^ AMP = ampicillin, CIP = ciprofloxacin, NAL = nalidixic acid, GEN = gentamicin, STR = streptomycin, FF = florfenicol, TET = tetracycline, CS = colistin, SU = sulfamethoxazole, TM = trimethoprim, CM = chloramphenicol, KAN = kanamycin, CTX = cefotaxime, CAZ = ceftazidime.

The association between AMP resistance, treatment group membership and MDR was tested with logistic regression analysis. The odds of an *E*. *coli* isolate being MDR was 27.6 times higher for AMP-resistant than for AMP-susceptible isolates (OR 27.6, IC95% [9.47, 80.75], *P* < 0.001), but MDR-resistant isolates were equally distributed between the two groups. Further, a statistically significant association was recorded between resistance to AMP and resistance to CIP (*P* < 0.01), STR (*P* < 0.001), TET (*P* < 0.001), SU (*P* < 0.001) and TM (*P* < 0.001).

One *E*. *coli* isolate, originating from the control group, was resistant to colistin (MIC 4 mg/l) and two *E*. *coli* isolates had a colistin MIC of 2 mg/l (current EUCAST ECOFF value). They were screened for the presence of mcr-1 gene using PCR, but yielded negative results.

## Discussion

The administration of amoxicillin for new-born piglets had no effect on the prevalence of hernias and abscesses in umbilical area of pigs until four weeks and in inguinal area until nine weeks of age. However, in contrast to our hypothesis, the treatment decreased the prevalence of umbilical hernias and abscesses at nine weeks of age. The amoxicillin treatment also reduced mortality and the occurrence of leg problems until weaning and increased growth of the pigs until the age of nine weeks. However, the prevalence of umbilical hernias and need for treatments was low in the herd studied. The treatment did not have an effect on the resistance pattern of the *E*. *coli* isolates from the faeces of the weaned pigs, but it is notable that we found a remarkable proportion of resistant and multi-resistant bacteria in the study herd.

Our general finding that the prevalence of umbilical hernias was evidently low when compared with other reports [1, 2] could be because we were able to follow the animals only up to the age of approximately nine weeks. It has been reported that hernias in pigs appear commonly at the age of 9–14 weeks [1]. Therefore, our results might underestimate the prevalence of hernias and it is debatable whether the final outcome would have been different had we been able to follow the pigs over a longer period. Nevertheless, in the present study, it is noteworthy that there was no significant difference in the size of hernias or abscesses between antimicrobial treated and non-treated pigs until the age of nine weeks, and that about half of the hernias or abscesses diagnosed at four weeks of age had disappeared at nine weeks of age. We stress the importance of diagnosis by palpation. Visual inspection might not be sufficient to distinguish between hernias and abscesses because shapes of abscesses can be the same as those of hernias.

There are only few studies concerning postnatal umbilical infections. In general, *E*. *coli*, *S*. *hyicus* and *Enterococcus* spp. were the most common bacterial species involved in umbilical infections [22], and *Trueperella pyogenes*, staphylococci and streptococci were found in connection with abscesses in pigs [23]. It is likely that in the present study the use of a single high dose of amoxicillin for newborn piglets prevented or treated the early umbilical infections occurring immediately after birth and therefore the prevalence of umbilical hernias was lower in treated piglets at the age of nine weeks. Agerso and Friis [24] have shown that after intramuscular injection of long-acting amoxicillin a plasma concentration of approximately 0.3 μ/ml was reached for about 48 hours [24], which was therapeutically sufficient when using the mean MIC-values for the pathogens commonly involved in infections in pigs [25]. However, other studies [1, 8, 5] reported failure to prevent umbilical disorders using prophylactic antimicrobial agents. It might be simply inappropriate to generalise the results achieved in one herd, including our study, because other factors could affect the results. For instance, the standard level of hygiene or previous use of antimicrobial agents in the herd remains unknown. The high level of antimicrobial resistance in the weaned pigs in our study might reflect that the study herd had been subject to several antimicrobial treatments already before the study period and this had exerted a longer-lasting selection pressure for resistant bacteria. Furthermore, in the study herd we were unable to speculate on the percentage of assisted farrowing by the farm personnel, even though it is known that abnormal stretching, possibly induced by birth intervention during farrowing, could be a factor in developing hernias [2].

Inguinal hernias in neonates are known to result mainly from genetic factors [5, 6, 26]. The etiology of inguinal hernias does not involve an infection and therefore antimicrobial agents have not been used in their prevention. The current findings that the treatment did not influence the prevalence of hernias or abscesses in inguinal areas of the pigs until the age of nine weeks may indeed support this case. Meanwhile, 0.1% of the prevalence of the inguinal hernias or abscesses in the present study was considerably low when compared with other studies (0.7% by Straw et al. [2]).

Searcy-Bernal et al. [1] reported that bacterial infection of the umbilical stump, mainly caused by an unhygienic environment, might also lead to failure in closing the umbilical cord. On that basis, Reiman et al. [10] demonstrated that the use of antibiotics for newborn piglets contributed to a decrease in the incidence of umbilical hernias together with a decrease in navel infections in weaning and finishing pigs. Therefore, a hygienic environment and disinfection of the wound shortly after birth or castration might help in reducing such bacterial infections. For the weaned pigs in the current study, however, the level of pen hygiene was not associated with the incidence of hernias or abscesses. Many studies suggested that hernias in neonates are more likely to originate at birth [1, 3]. Hence, a hygienic environment per se or disinfection treatment may contribute to preventing hernias to a greater extent for new-born than for weaned pigs. However, further studies are needed to demonstrate a causal link between sanitary conditions and the prevalence of hernias in pigs of different ages.

Our finding that hernias might not be a significant cause of reduction in growth rate in nine week old pigs is supported by the results of Searcy-Bernal et al. [1]. Straw et al. [2], however, reported that development of hernias may be associated with slower growth rate in growing pigs, and also could be related to higher mortality rate in finisher pigs. Diverse consequences from those studies could probably be due to the different examination time points for detecting defects or measuring growth performance.

In the present study, piglets in the antibiotic treatment group grew faster and had lower mortality than those in the untreated group. Several studies have demonstrated that many different classes of antimicrobials added at sub-therapeutic levels in the diets can lead to improved growth rate and reduced mortality in weaned pigs [27, 28, 29]. Furthermore, the efficacy of antibiotic treatment on growth performance could be even greater under conditions of substandard hygiene [27]. For such reasons, several antimicrobial agents as growth promoters have been widely used in pig production in the past despite the ever-increasing problem of antimicrobial resistance [30]. In those studies, however, antimicrobial agents were administered at the same time as growth was measured, whereas in this study a single dose was administered about a month before the growth was measured. There is therefore no clear explanation for our finding of greater growth in the treated group. In Finland, as in other EU countries, however, the use of antimicrobial agents is not allowed for the purpose of growth promotion, and is also not recommended for preventing diseases.

Leg problems are common in suckling piglets in modern pig farming and are generally known to be caused by infected foot and skin lesions [31], or by septicaemic infection at or soon after birth [23]. One of the entry routes for the infection is through an infected umbilicus. Therefore, our findings make sense because if the treatment prevented umbilical infections, it also consequently decreased the need to treat leg problems. Zoric et al. [32] showed that penicillin administration for lame suckling piglets was effective in treating leg problems. In contrast to our expectations, however, we found no difference in the need for medication to treat other diseases in the piglets. Meanwhile, several studies demonstrated that administration of antimicrobials to newborn piglets could disturb normal development of microbiota for an extended period of time [13, 33]. The microbiota plays an important role in the health of piglets, and its development could be affected by several factors, including antimicrobial administration [34, 35, 36, 37]. Considering the potential alterations in the gut microbiota, therefore, it should be considered whether prophylactic treatments for young animals are worthwhile.

In previous studies, treatment regimens for pigs with AMP given over several days have been associated with increases in the proportions of AMP-resistant *Enterobacteriacae* or *E*. *coli* [17, 38]. In the present study, the single one-day dosing regimen and four week temporary lag between dosing and sampling might be the reason why we did not record a significant difference in the proportions of AMP-resistant coliform bacteria between the groups. Furthermore, it might be also possible that faecal samples collected from the non-treated group contained faeces from single animals that were treated with antibiotics during the nursing period. The most important factor, however, is most likely the pre-existing antimicrobial resistant background coliform bacteria at the farm. Resistant bacteria probably persist and circulate at the farm and are introduced into the piglets from the environment because of the use of antimicrobial treatments of infectious diseases increasing selection pressure of resistance genes. The most predominant single resistance pattern in *E*. *coli* isolates was AMP-STR-TET-SU-TM. Resistance against these antimicrobial agents has been common also in several studies performed on pig farms in other countries [39, 40, 41] as well as in the EU monitoring of indicator *E*. *coli* from pigs at slaughter [16]. This pattern was also previously associated with multiple drug-resistance-conferring plasmids [42]. In the present study, this profile is most likely not only due to the use of antimicrobials on the farm, but indicates the presence of resistance-conferring mobile genetic elements in the porcine intestinal microbiome. This is supported by the associations between AMP-resistance and resistance to STR, TET, SU and TM, and as AMP-selection at isolation appeared also to select for other resistance traits. Previous studies have indicated that continuous low-dose use of antibiotics to livestock favours propagation and selection of large number resistance genes [43]. Increased selection of resistance is more strongly associated with the high number of single animals treated than to the total amount of antibiotic used [44]. Continuous low-dose treatment is also associated with the selection of multidrug resistance [43]. Therefore, prudent use of antimicrobials and proper sanitary management are highly recommended to eradicate or decrease the proportions of resistant bacteria on the farm. However, based on the susceptibility of all the tested *E*. *coli* isolates to cefotaxime and ceftazidime, no extended spectrum betalactamase (ESBL)-producers were detected despite continuous use of amoxicillin on the farm. This could indicate that ESBL encoding genes are not present in the farm microbiome.

## Conclusions

Contrary to the hypothesis of the study, administration of one dose of amoxicillin during the first day of life decreased the prevalence of umbilical hernias and abscesses at nine weeks of age and the need to treat the piglets for leg problems. Moreover, the treatment increased the growth of the pigs between birth and nine weeks of age, and decreased mortality before weaning. However, the effects on the prevalence of umbilical hernia as well as on growth were minor compared to the workload of the treatment. On the other hand, the treatment had no effect on the prevalence of inguinal hernias and abscesses until the age of nine weeks. The size of hernias and abscesses in the umbilical area, or the need to treat the piglets for other diseases during suckling, was likewise not affected by the treatment. Although we found no difference in the prevalence of antimicrobial resistant intestinal coliform bacteria between the groups, the current results need to be interpreted with caution as the most likely reason lies in the presence of background resistance among intestinal coliform bacteria and *E*.*coli*. Repeated long term antibiotic treatment of large number of piglets during their first day of life could have an additive effect promoting the selection of resistance genes at the farm. Summarizing, these results showed that the single-dose antibiotic treatment at birth prevented umbilical defects, promoted growth and decreased mortality at nine weeks of age, but only to limited extent. Therefore, we did not find enough evidence to support the practice of preventive use of antibiotics for newborn piglets, especially considering the possible influence on the development of the normal intestinal microbiota and the selection of antimicrobial resistance genes at the herd level. Furthermore, the overall costs of the antimicrobial treatments are likely to exceed the observed minimal benefits.
